# The Histological, Effectoromic, and Transcriptomic Analyses of *Solanum pinnatisectum* Reveal an Upregulation of Multiple *NBS-LRR* Genes Suppressing *Phytophthora infestans* Infection

**DOI:** 10.3390/ijms21093211

**Published:** 2020-05-01

**Authors:** Biao Gu, Xiaoli Cao, Xiaoli Zhou, Zhaodan Chen, Qinhu Wang, Wei Liu, Qin Chen, Hua Zhao

**Affiliations:** 1 State Key Laboratory of Crop Stress Biology for Arid Areas, Northwest A&F University, Yangling 712100, China; bgu@nwafu.edu.cn (B.G.); 17110700034@fudan.edu.cn (X.C.); zhou18392117436@163.com (X.Z.); 2018102039@njau.edu.cn (Z.C.); wangqinhu@nwafu.edu.cn (Q.W.); wliu@nwsuaf.edu.cn (W.L.); 2 College of Plant Protection, Northwest A&F University, Yangling 712100, China; 3 College of Agronomy, Northwest A&F University, Yangling 712100, China; 4 College of Food Science and Engineering, Northwest A&F University, Yangling 712100, China

**Keywords:** wild potato, late blight, resistance genes, histological features, avirulence gene screen, RNA-seq analysis

## Abstract

Utilization of disease resistance components from wild potatoes is a promising and sustainable approach to control *Phytophthora* blight. Here, we combined avirulence (*Avr*) genes screen with RNA-seq analysis to discover the potential mechanism of resistance in Mexican wild potato species, *Solanum pinnatisectum*. Histological characterization displayed that hyphal expansion was significantly restricted in epidermal cells and mesophyll cell death was predominant, indicating that a typical defense response was initiated in *S. pinnatisectum*. Inoculation of *S. pinnatisectum* with diverse *Phytophthora infestans* isolates showed distinct resistance patterns, suggesting that *S. pinnatisectum* has complex genetic resistance to most of the prevalent races of *P. infestans* in northwestern China. Further analysis by *Avr* gene screens and comparative transcriptomic profiling revealed the presence and upregulation of multiple plant *NBS-LRR* genes corresponding to biotic stresses. Six *NBS-LRR* alleles of *R1*, *R2*, *R3a*, *R3b*, *R4*, and *Rpi-smira2* were detected, and over 60% of the 112 detected NLR proteins were significantly induced in *S. pinnatisectum*. On the contrary, despite the expression of the *Rpi-blb1*, *Rpi-vnt1*, and *Rpi-smira1* alleles, fewer NLR proteins were expressed in susceptible *Solanum cardophyllum*. Thus, the enriched *NLR* genes in *S. pinnatisectum* make it an ideal genetic resource for the discovery and deployment of resistance genes for potato breeding.

## 1. Introduction

Potatoes, one of the most important food crops, have been affected by late blight disease for nearly 180 years [1]. To decrease the yield losses caused by *Phytophthora infestans*, cultivars of *Solanum tuberosum* expressing diverse resistance (*R*) genes have been developed and extensively applied in potato growing areas worldwide. Many typical *R* genes, such as *R1* to *R11* from *Solanum demissum*, were initially demonstrated to be successful in improving disease-resistant commercial potatoes. However, the *R*-gene-insensitive strains of *P. infestans* frequently emerged after large-scale application of resistant cultivars harboring major *R* genes [2,3]. For instance, more aggressive *P. infestans* isolates escaping recognition by *R1*-*R11* genes were reported worldwide [4,5]. However, the outstanding disease-free phenotypes and long-term stability of *R* genes remain valuable, stimulating numerous attempts to discover natural immune receptors from *Solanum* species for potato resistance breeding [6,7].

Over 1000 accessions of 216 wild *Solanum* spp. throughout North and South America were investigated as potential enriched genetic resources for molecular and genetic dissection of the resistance genes against *P. infestans* [8]. To date, less than 70 *R* genes or alleles conferring resistance to *P. infestans* (*Rpi*) have been identified, mapped, and cloned from several wild *Solanum* species, such as *S. berthaultii*, *S. bulbocastanum*, *S. demissum*, *S. microdontum*, *S. mochiquense*, *S. stoloniferum*, and *S. venturii* [8,9]. Most of the *Rpi* genes against potato late blight are members of the nucleotide-binding site and leucine-rich repeat (NLR) protein family [9,10]. Many *Rpi* genes have been defeated by fast-evolving *P. infestans* isolates, but both laboratory and field tests demonstrated that pyramiding several *Rpi* genes into a single potato cultivar was effective in preventing the *R*-gene destroyer *P. infestans* [11]. As a result, more efforts and research tools are still needed to facilitate the identification and rational deployment of resistant genes.

In addition to the techniques associated with high-throughput sequencing to discover resistance traits in plants, *In planta* screens of *Phytophthora* RxLR effectors is an alternative genomic approach for the detection of race-specific defense responses. These effector probes could accelerate *R*-gene identification, distinguish functional redundancy, detect recognition specificity, optimize *R*-gene utilization, and avoid sexual incompatibility in the traditional map-based genetic analysis [12]. A functional homolog of the *RB*/*Rpi-blb1* gene from a non-crossable variety of *S. bulbocastanum* was mined by PVX (Potato Virus X) agroinfection with AVRblb1 in *Nicotiana benthamiana* [13]. The recognition between AVRblb1 and a RB functional allele in crossable *S. stoloniferum* enabled efficient introgression of *Rpi-sto1* into cultivated potato-breeding materials [14]. Both qualitative *Rpi* genes (*R3a*, *R3b*, *R4*, and *Rpi-Smira1*) and a quantitative *Rpi* gene (*Rpi-Smira2*) in the potato cultivar ‘Sarpo Mira’ were identified via effectoromics as well [15].

To conquer fast-evolving *P. infestans*, a continuous influx of new *Rpi* genes with broad-spectrum resistance from genetically diverse wild germplasm are being pursued by potato breeders [12,16,17]. One wild Mexican diploid (2×) *Solanum* species *S. pinnatisectum* with an endosperm balance number of 1 (1EBN) has gained attention because it showed high-level resistance to late blight, and is crossable with another Mexican 2× (1EBN) *Solanum* species, *S. cardiophyllum*, which is susceptible to *P. infestans* [18,19,20]. So far, two *Rpi* genes, *Rpi1* and *Rpi2*, have been characterized and mapped to chromosome 7 of *S. pinnatisectum* [20]. Analyses by Park et al. [21] indicated that gene clusters for late blight *Rpi* are prevalent on chromosomes 4 and 11 of *S. pinnatisectum*, In addition, *Rpi1*, a quantitative trait loci (QTL) on chromosome 7 for resistance to *P. infestans* (Pi_QTL), was recorded in the offspring of *S. phureja* and *S. tuberosum* [22]. Besides, the PiAvr2 recognition *R* gene was also detected via allele-mining [23]. Therefore, *S. pinnatisectum* was considered to be a valuable wild *Solanum* resource for the exploitation of *Rpi* genes.

In this research, based on the histological features of the resistant reaction, we combined a stripped-down version of the *Avr* gene screen with RNA-seq analysis to dissect the potential resistance machinery of *S. pinnatisectum*. The remarkable defense responses began at 6 h post-inoculation. Based on the RNA-seq samples together with the histological features, over 60% of 112 NLR protein candidates were responsible for *P. infestans* resistance, including the known *Rpi* genes *R1*, *R2*, *R3a*, *R3b*, *R4*, and *Rpi-smira2*. Compared to the *NLR* genes expressed in the susceptible cultivar *S. cardiophyllum*, a distinct group of *NLR* genes with higher expression levels were clustered in *S. pinnatisectum*. These results provide useful resources for guiding the identification and rational application of *Rpi* genes in the resistance breeding of potatoes.

## 2. Results

### 2.1. Infection with P. infestans on S. pinnatisectum is Evident

**Figure 1:**
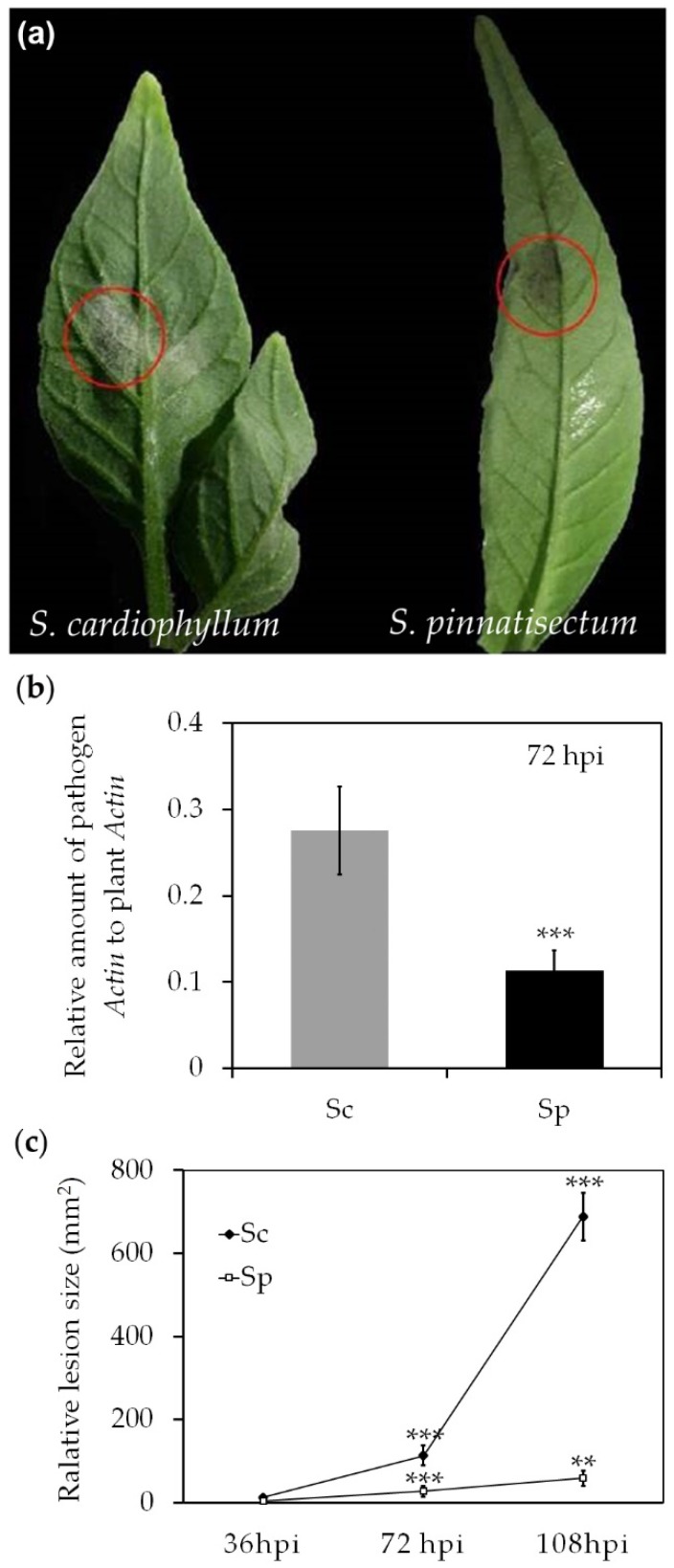
Infection of *Solanum pinnatisectum *and *Solanum cardiophyllum* by *Phytophthora infestans*. (**a**) The symptom on *S. cardiophyllum* and *S. pinnatisectum* 72 h post-inoculation (hpi). (**b**) The biomass of *P. infestans* detected in *S. pinnatisectum* and *S. cardiophyllum* 72 hpi. Each error bar represents the mean ± SD. (**c**) The lesion size on *S. pinnatisectum* and *S. cardiophyllum* measured at different times post inoculation. Sc: *S. cardiophyllum*, Sp: *S. pinnatisectum*. (Each error bar represents the mean ± SD, ** and *** indicate statistical significance where *p* < 0.05 and *p* < 0.001 using t test, respectively).

In order to determine the susceptibility of the wild potato species *S. pinnatisectum* to infection by *P. infestans*, zoospore inoculation was performed with an aggressive isolate, Pi21366, which is virulent to all tested potato *R*-gene differentials (R1-R11). The detached leaves of the susceptible species *S. cardiophyllum* and resistant species *S. pinnatisectum* were inoculated with 10^3^ zoospores. As shown in Figure 1a, only restricted water-soaked lesions developed at 72 h post inoculation (hpi) on *S. pinnatisectum* leaves. Well-expanded diseased areas with fluffy hyphae and a large number of sporangia were observed on the leaf surface of *S. cardiophyllum* (Figure 1a). The relative biomass of *P. infestans* measured at 72 hpi via qPCR was more than 2-fold greater in diseased tissue on *S. cardiophyllum* than that detected on leaves of *S. pinnatisectum* (Figure 1b). At the later stage of infection (108 hpi), the lesions expanded to the whole *S. cardiophyllum* leaves, but lesions on *S. pinnatisectum* were only slightly larger than at 72 hpi (Figure 1c).

### 2.2. Histological Characteristics of S. pinnatisectum Infected by P. infestans

**Figure 2:**
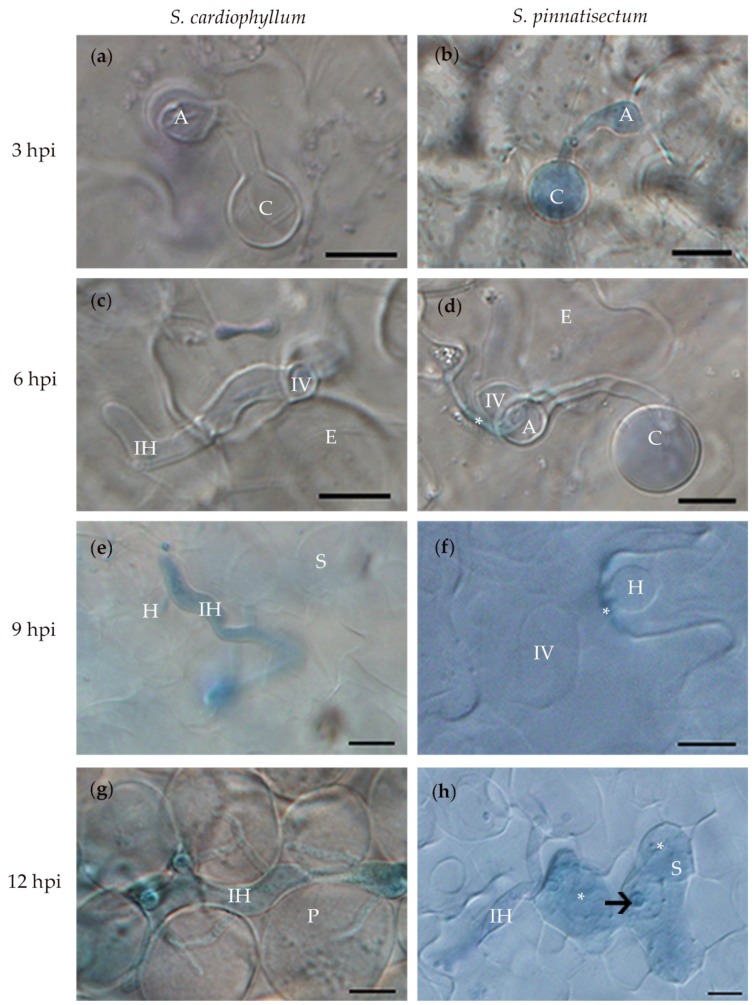
Microscopic characterization of *Solanum pinnatisectum* and *Solanum cardiophyllum* interacted with *Phytophthora infestans* at different times post-inoculation. (**a**) and (**b**), the germinated cysts with appressorium at the tip on the leaf surface of *S. cardiophyllum* and *S. pinnatisectum* 3 h post-inoculation (hpi), respectively. (**c**) and (**d**), the pathogen penetrated into the epidermal cell of *S. cardiophyllum* and *S. pinnatisectum* 6 hpi, * in (d) indicates cell wall deposition at the infection site of epidermal cell on *S. pinnatisectum*. (**e**) Intercellular hyphae with haustoria invade spongy cells of *S. cardiophyllum* 9 hpi. (**f**) The pathogen extends underneath the cuticle as the infection vessel and forms haustoria in the epidermal cells of *S. pinnatisectum* 9 hpi. (**g**) Intercellular hyphae expand with multiple branches in the palisade cells of *S. cardiophyllum* 12 hpi. (**h**) Penetration of intercellular hyphae is stopped by the necrosis of mesophyll cells on *S. pinnatisectum* 12 hpi; the haustorium is pointed by the arrow; and the deeply blue-stained tissues indicate cell necrosis (indicate as *). A: appressorium, C: cyst, E: epidermal cell, H: haustorium, IH: intercellular hyphae, IV: infection vessel, P: palisade cell, S: sponge cell. Bar = 10 μm.

To investigate the histological characteristics of early interactions between *P. infestans* and *S. pinnatisectum* or *S. cardiophyllum*, detached leaves were drop inoculated with zoospores and collected at 3, 6, 9, and 12 hpi, respectively. Within 3 hpi, no significant difference was observed between *S. pinnatisectum* and the susceptible control *S. cardiophyllum*. More than 80% of the cysts germinated on the leaf surface of both species, and most of them produced an appressorium at the tips of the germ tubes (Table 1, Figure 2a,b; both Table 1 and Figure 2 were modified from Cao [24]). Invasive hyphae were observed as well, which were predominant at the anticlinal walls between the epidermal cells (Figure 2a,b). By 6 hpi, infection vesicles were observed beneath appressoria in the epidermal cells of both *S. pinnatisectum* and the susceptible control (Figure 2c,d). The intercellular hyphae were generated and extended intercellularly among spongy cells, although penetration into the spongy tissue of *S. pinnatisectum* was occasionally visible (Table 1). By this time point, the reaction of plant cells and cell wall deposition were universal in the epidermal cells of *S. pinnatisectum* in response to the infection of *P. infestans* (Figure 2d) but not in the epidermal cells of *S. cardiophyllum* (Figure 2c).

Phenotype differences between *S. pinnatisectum* and *S. cardiophyllum* became more evident at 9 hpi. In *S. pinnatisectum*, more than 90% of the infection hyphae were confined to the site of the initial penetration of the epidermal cell, while in *S. cardiophyllum*, the infection hyphae extended rapidly, with more than 40% of the hyphae reaching spongy tissue, and the haustoria were visible (Table 1, Figure 2e,f). In *S. pinnatisectum*, the infection was still restricted to the epidermal cells by 12 hpi, and some epidermal cells and mesophyll cells beneath the infection site were necrotic (Figure 2h). By this time point, nearly 60% of the hyphae spread into the spongy tissue of *S. cardiophyllum*, and about 25% of the hyphae had invaded into the palisade tissue but necrosis was rarely observed (Table 1, Figure 2g). These results provided preliminary evidence that a typical defense response rather than cell wall-associated penetration resistance was initiated in *S. pinnatisectum* upon *P. infestans* inoculation. Based on the above microscopic observations, we determined 3, 9, and 12 hpi as time points to investigate the transcriptional dynamics of *S. pinnatisectum* compared to *S. cardiophyllum*.

### 2.3. S. pinnatisectum Reveals Complicated Pathotypes Based on Multiple R Genes

**Figure 3:**
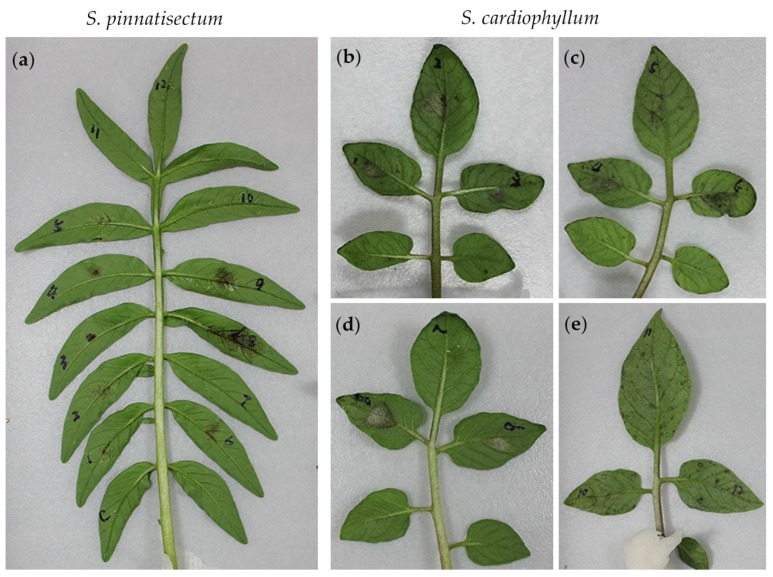
Symptoms on *Solanum pinnatisectum* and *S. cardiophyllum* leaves inoculated with 12 isolates of *Phytophthora infestans* 4 days post-inoculation. (**a**) Symptoms on a leaflet of *S. pinnatisectum* inoculated with 12 isolates of *P. infestans*. (**b**) to (**e**) Symptoms on leaflets of *S. cardiophyllum* caused by 12 isolates of *P. infestans*. C: water control. The virulence spectrum of these 12 *P. infestans* isolates are described in Table 2.

To dissect the genetic resistance of *S. pinnatisectum*, the detached leaves were subjected to inoculation with a panel of 12 *P. infestans* isolates displaying SSR-based genotype variations [5] and *R*-gene-dependent pathotype diversity (Table 2). The phenotypes of both *S. pinnatisectum* and *S. cardiophyllum* to each isolate were evaluated macroscopically and qualitatively based on a hypersensitive response (HR) or expanding sporulating lesions. As shown in Figure 3a, isolate 10, 11, and 12, and H_2_O control failed to generate any visible lesions, whereas isolate 1, 2, and 5 produced many tiny brown spots on *S. pinnatisectum*. Lesions caused by isolate 3, 4, and 6 were restricted to inoculation sites, but isolate 8 and 9 were more virulent on the inoculation sites of *S. pinnatisectum* (Figure 3a). Based on the pathotypes of isolate 8 and 9, functional homologs of R1, R2, R5, R8, R9, R10, and R11 may not be present in *S. pinnatisectum* or are not sufficient to trigger defense responses. On the contrary, only isolate 7 was avirulent on *S. cardiophyllum* (Figure 3d). Ten other isolates, except isolate 5, were more aggressive on the susceptible control *S. cardiophyllum* (Figure 3b–e). Interestingly, hypervirulent isolate 12, escaping the recognition of all 11 *R* genes *S. demissum*, and the isolate 6, which is avirulent on most of the R1–R11 differential hosts, produced similar pathotypes on *S. pinnatisectum* and *S. cardiophyllum* (Figure 3a,c). Moreover, we noticed that isolates 8 and 9, showing avirulence on R5, R8, and R9 differential hosts, were virulent on *S. pinnatisectum*. However, isolate 6, showing avirulence on R5, R8, and R9 differential host as well, was avirulent on *S. pinnatisectum*. Altogether, the distinct resistance patterns observed in the detached-leaf assay illustrate that the resistance machinery in *S. pinnatisectum* is complicated and no single *R* gene of R1–R11 determines the avirulence phenotype.

### 2.4. RxLR Effector Screening Reveals Different R Protein Composition in S. pinnatisectum and S. cardiophyllum

**Figure 4:**
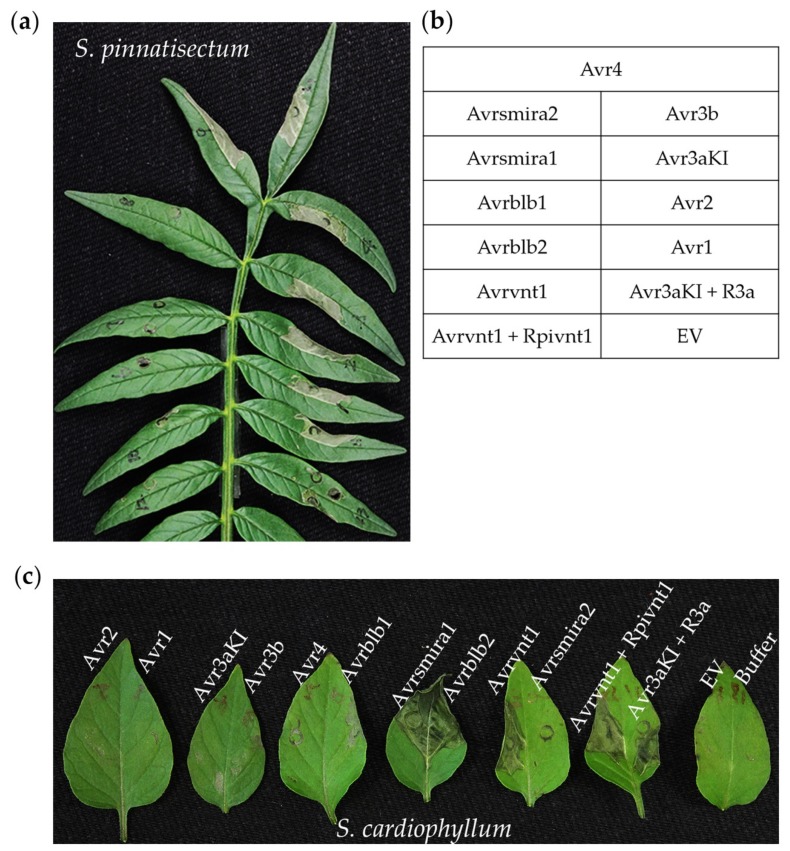
Detection of cell death on *Solanum pinnatisectum* and *Solanum cardiophyllum* triggered by 10 cloned notable *Avr* genes and *Avr*-*R* gene pairs of *Phytophthora infestans*. Avrvnt1/Rpi-vnt1 and Avr3aKI/R3a were used as positive, buffer or empty vector (EV) was used as the negative control. Photographs were taken 3–4 days after agroinfiltration (**a**) Cell death on the leaflet of *S. pinnatisectum* caused by Avrsmira2, Avr4, Avr3b, Avr3aKI, Avr2, and Avr1. (**b**) Agroinfiltration sites with the matching *Avr* gene or *Avr-R* gene pair on each pinnately compound leaf. (**c**) Cell death on leaves of the susceptible cultivar *S. cardiophyllum* triggered by Avrsmira1, Avrblb2, and Avrvnt1.

Ten cloned notable RxLR effector genes (*Avr* genes) and two *Avr*-*R* gene pairs were delivered into *S. pinnatisectum* or *S. cardiophyllum* leaves by agroinfiltration. Based on the development of cell death symptoms, it is clear that *S. pinnatisectum* and *S. cardiophyllum* possess distinct recognition spectra of the *Avr* genes tested (Figure 4, Supplementary Table S1). Of the 10 tested *Avr* genes, 6 of them, *Avr1*, *Avr2*, *Avr3aKI*, *Avr3b*, *Avr4*, and *Avrsmira2*, triggered hypersensitive responses in *S. pinnatisectum* (Figure 4a,b), whereas in *S. cardiophyllum*, cell death was observed on leaves expressing *Avrblb2*, *Avrsmira1*, and *Avrvnt1* (Figure 4c). The two potato species have distinct matching *R* genes, all of which belong to typical *NBS*-*LRR* resistance genes. The higher number of *R* genes in *S. pinnatisectum* may be responsible for its strong resistance to diverse isolates of *P. infestans*. *Avr3aKI* and *Avr3a*-*R3a* both triggered cell death in *S. pinnatisectum*, but functional *R3a* was absent in *S. cardiophyllum*. It is remarkable that neither *Avrvnt1* nor the *Avrvnt1*/*Rpivnt1* pair produced cell death in *S. pinnatisectum*, indicating that the presence of a specific interacting protein rather than a functional homolog of *Rpi-vnt1* is needed for the initiation of HR.

More interestingly, this effector-based avirulence pattern does not perfectly match the inoculation pathotypes. For instance, *R1* and *R2* functional alleles were detected via the effector infiltration assay in *S. pinnatisectum*, and some *Avr1*- or *Avr2*-expressing isolates still caused infection whereas some *Avr1*- and/or *Avr2*-expressing isolates did not (Figure 4a, Table 2). Similarly, even though *Rpi-blb2*, *Rpi-smira1*, and *Rpi-vnt1* are expressed in *S. cardiophyllum* (Figure 4c) and the expression of some cognate *Avr* genes was validated in several tested isolates [25], 11 out of the 12 tested *P. infestans* isolates were more aggressive on this wild potato species (Table 2). These results raise the possibility that pathogens deliver multiple effectors to suppress effector-triggered immunity (ETI) and induce potato susceptibility.

### 2.5. Transcriptome Dynamics of NLR Genes in Susceptible and Resistant Potatoes against P. infestans

According to the previous histological observations in susceptible and resistant cultivars upon *P. infestans* challenge, four key time points were established at the early stage of infection. Inoculated leaves of *S. cardiophyllum* or *S. pinnatisectum* were collected at 6, 9, and 12 hpi, and samples at 0 hpi were used as the control, respectively. Illumina sequencing was conducted to generate transcriptome profiles of both the pathogen and potato over the infection time course. A summary of the number of potato reads after sequence filtering to remove short-length and low-quality sequences is shown in Supplementary Table S2. Approximately 31–70% of the sequences from each sample were mapped to the *S. tuberosum* genome.

**Figure 5:**
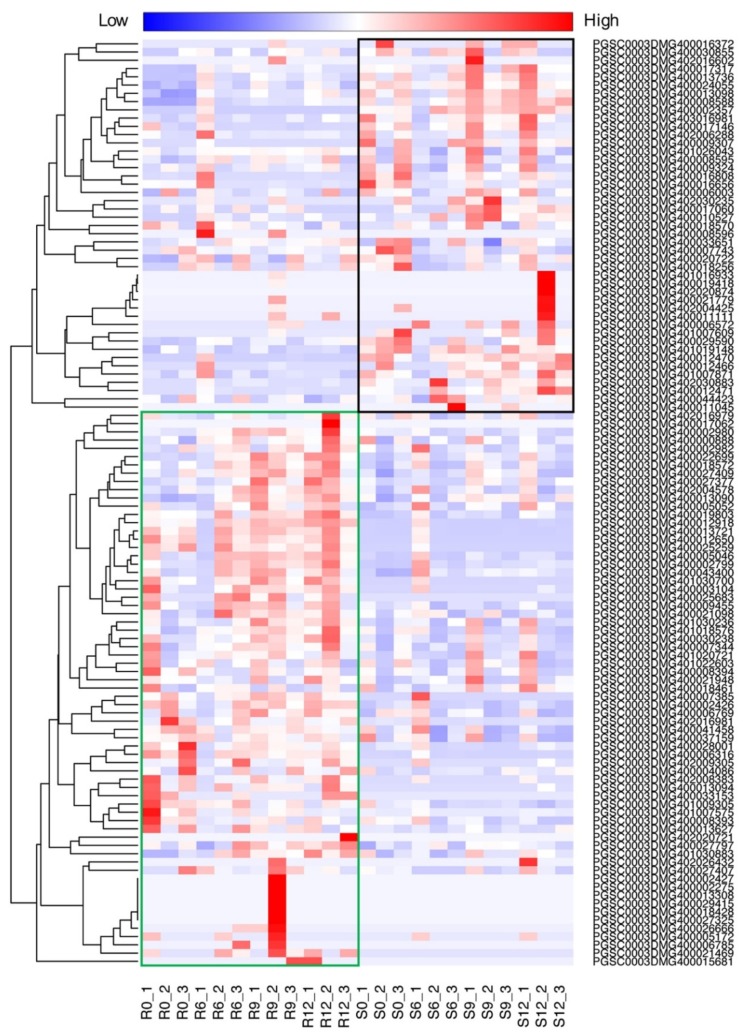
Expression profile of putative nucleotide-binding site and leucine-rich repeat (NLR) orthologous genes in infected *S. pinnatisectum* and *S. cardiophyllum* at 0, 6, 9, and 12 hpi. The heatmap indicates the sum of counts per million (CPM) values for genes in subclasses; the data are in Supplementary Material file S1. The green rectangle frame harbors most upregulated *NLR* genes in *S. pinnatisectum*. The black rectangle frame includes the most upregulated *NLR* genes in *S. cardiophyllum*. Each time point contains three replicates. R, refers to *S. pinnatisectum*; S, refers to *S. cardiophyllum*.

To characterize the molecular basis of the defense mechanism induced after pathogen infection, we identified the *NLR* genes (see the materials and methods) from the potato genome and investigated their expression. A total of 123 *NLR* genes were annotated. Of these, 112 have at least one read in at least one sample. Clustering of the expression profiling of the *NLR* genes revealed that they could be divided into two groups related to the resistant (Sp) and susceptible (Sc) potato cultivar, respectively (Figure 5). Previous studies showed that *Rpi1* and *Rpi2* have been mapped to chromosome 7 of *S. pinnatisectum* [20]. However, according to our RNAseq data, only two NLRs, PGSC0003DMG401030700 and PGSC0003DMG400017317, were dramatically upregulated in the chromosome 7 region of *S. pinnatisectum* and *S. cardophyllum*, respectively. In general, over 60% of NLRs were induced and exhibited higher expression levels in *S. pinnatisectum* but not in *S. cardiophyllum* after *P. infestans* infection. In particular, R1 homologue (PGSC0003DMG402004578), R2 homolgue (PGSC0003DMG400025259), and R3a-like genes (PGSC0003DMG400009455, PGSC0003DMG400018570, and PGSC0003DMG400027377) were identified in the upregulated *NLRs* of the Sp group. Thus, the massive expression of *NLR* genes in *S. pinnatisectum* is probably responsible for the resistant phenotype of *S. pinnatisectum*. While most of the *NLR* genes coinciding with *S. pinnatisectum* resistance are highly expressed at 12 hpi, some of them are expressed immediately upon infection (Figure 5), indicating that *S. pinnatisectum* may recruit different NLRs at different stages to deal with the pathogen attack.

## 3. Discussion

Compared to other wild potato species, very few novel *R* genes or functional homologues have been identified from *S. pinnatisectum* in the last two decades, but this Mexican wild potato species has been proposed to contain potential qualitative and quantitative resistance against *P. infestans*. Kuhl et al. [18] characterized 13 accessions of *S. pinnatisectum* by means of *P. infestans* zoospore inoculation, and more than 10 of them showed resistance, including accession PI275233. A large proportion of 44 *S. pinnatisectum* accessions were reported to be resistant to *P. infestans* in laboratory assays and in field sprays [8]. In agreement with the above results, our studies verified that *S. pinnatisectum* displayed considerable resistance against multiple *P. infestans* isolates, which are characterized by different SSR genotypes and diverse pathotypes [5]. Especially, those predominant isolates, which are virulent to the R1–R11 potato differential set, failed to infect this wild potato species. On the contrary, these supervirulent isolates induced rapidly expanding water-soaked lesions on susceptible cultivars of *S. cardiophyllum*. Remarkable resistance or tolerance against *P. infestans* was observed from 3 to 72 hpi, and much lower amounts of inoculum proliferated in the diseased tissue of *S. pinnatisectum*.

The interaction between Avr and R protein triggered immediate and strong hypersensitive responses (HRs) in plant cells, which led to lesion-free phenotypes or could be visible as localized cell death [26]. The responses of plants to pathogen stress were more obvious and pervasive in mesophyll cells within 9 to 12 hpi but were rare in the susceptible control, *S. cardiophyllum*. Necrosis was visible in the infected mesophyll cells of *S. pinnatisectum* at 12 hpi and later, indicating that the HR was induced upon infection. As a result, expansion of *P. infestans* was restricted to the epidermis, consistent with other resistant germplasm of potato [27]. Significant variations of histological characteristics in the infection processes between *S. pinnatisectum* and the susceptible control *S. cardiophyllum* also illustrates that defense responses are different from penetration resistance as cysts normally geminated and penetrated into epidermal cells before 6 hpi. Thus, the *R* gene-mediated ETI probably plays a key role in disease resistance and tolerance under *P. infestans* challenge. The RNA-seq data verified this hypothesis by revealing an upregulation of many ETI components.

To sketch the genetic resistance of *S. pinnatisectum*, we first revaluated the late blight resistance of *S. pinnatisectum* by using different *P. infestans* isolates with distinct pathotypes. Surprisingly, only two isolates (No. 8 and 9) could readily infect *S. pinnatisectum*, indicating that the expression of *Avr1* and *Avr2* did not affect the infection. Supervirulent dominant isolates (No. 12) rarely infected this resistant cultivar but displayed high aggressiveness on *S. cardiophyllum*. Pathotype association analysis suggests that multiple genes govern the resistance. The use of *P. infestans* strains could be challenging, because multiple *R* genes in resistant cultivars can mask recognition specificities. Many resistance breeding studies have employed *Phytophthora* RxLR effectors rather than diverse *P. infestans* isolates as markers to mine novel *R* genes or dissect functional *R* gene alleles [28]. To better discriminate the specific *R* gene activities, in this research, we also took advantage of the RxLR effector screen and RNA-seq analysis and discovered a distinct group of *NLR* genes that are responsible for the remarkable resistance of *S. pinnatisectum*.

The functional homologues of qualitative *R* genes *R1*, *R2*, *R3a*, *R3b*, and *R4* and quantitative *R* gene *Rpi-smira2* could be accurately detected using *Avr1*, *Avr2*, *Avr3aKI*, *Avr3b*, *Avr4*, and *Avrsmira2* in *S. pinnatisectum*. The pathogenic variations of different *Avr1*- and *Avr2*-expressing isolates suggest that other potential *Avr* genes are involved in *R* gene recognition or ETI suppression. Such a special case is that the loss of resistance of Rpi-blb1 (RB) is caused by ETI suppressing the variant of Avrblb1 [29]. Thus, the detection of mutations in cloned *Avr* probe genes may not be sufficient for a virulent profile prediction of filed isolates. This is different from the scenario of *P. sojae*, in which the presence of functional *Avr* genes in selected isolates is highly consistent with their avirulent phenotype [30]. Moreover, co-expression of *Avrvnt1* and *Rpi-vnt1* or single *Avrvnt1* failed to induce HR in *S. pinnatisectum*, suggesting that specific interacting proteins are needed for Avrvnt1-Rpi-vnt1 recognition. As Gao C et al. pointed out [31], glycerate 3-kinase (GLYK) is involved in activation of of Rpi-vnt1. Similarly, Avrblb1 and Rpi-blb1 only triggered typical HR in *N. benthamiana* but not in *Arabidopsis thaliana*, indicating that species-specific interactors are involved in cell death induction [32]. According to the guard model and the decoy model, the Avr-R protein recognition specificity would depend on secondary molecules. For example, BSL1 was initially identified as a key chaperone for PiAvr2 and R2 interaction, and then was predicted to regulate multiple *R* gene-mediated immunity responses [33]. As a result, future studies should not only seek identification of novel genes conferring resistance but also patterns of defense genes activated in ETI.

None of those six *R* genes exist in *S. cardiophyllum*, but three other *R* genes (*Rpi-blb2*, *Rpi-vnt1*, and *Rpi-smira1*) were detected in this susceptible control by transient expression of cognate *Avr* genes. These *R* genes did not provide adequate resistance for this cultivar and resulted in high susceptibility. Currently, for late-blight management, breeders prefer to pyramid multiple *R* genes in one cultivar to avoid the rapid defeat of *R* genes [11]. Here, we have an opposite example, as although *Rpi-blb2* and *Rpi-vnt1* were documented as a broad-spectrum resistance gene from *S. bulbocastanum* and *S. venturi* [34,35], the natural combination of *Rpi-blb2*, *Rpi-vnt1*, and *Rpi-smira1* in *S. cardiophyllum* changed its susceptibility against most isolates used in our detached-leaf inoculation assay. Moreover, the mutation or silencing of cognate RXLR effectors may not be responsible for the loss of resistance, since the transcripts of conserved *Avrvnt1*, *Avrblb2*, and *Avrsmira1* were detected in at least one of isolate 1, 6, or 12 [25]. Thus, more effort is necessary for the construction of desired long-term stable resistant cultivars.

Despite only part of the known *Avr* genes being employed in the screening, the results suggest that more active *R* genes were clustered in *S. pinnatisectum*. It is also reported that even defeated *R* genes in wild potatoes may lead to a delay in infection. Considering that all tested local isolates of *P. infestans* contain truncated Avr4 [25], our findings suggest that the remarkable resistance of *S. pinnatisectum* is probably due to pyramiding of R1, R2, *R3a*, *R3b*, and *Rpi-smira2*. Of course, genetic validations are required to test whether the desired resistance can be attributed to these functional alleles. Then, the major genes for late blight resistance can be cis-transformed into potato cultivars. In addition, the degree of resistance is dependent on the genotype of the *P. infestans* population, and monitoring for virulence to specific *R* genes in the local *P. infestans* population can assist the deployment of *R* genes [36]. Once the transgenic techniques are broadly accepted, it could accelerate genetic modifications with *R* genes.

The RNA-seq data revealed that a massive number of immune-associated *NLR* genes, including a distinct *NLR* gene group, were dramatically upregulated in *S. pinnatisectum*. Despite the low similarity between different orthologs and some species-specific *NLR* genes being absent in our mapping process, the RNA profile analysis validated the previous effector screen and demonstrated the expression of multiple *NLR* genes. Considering that most of the known resistant genes belong to the *NBS-LRR* superfamily with a 3-kb average length [17], we mapped RNAseq reads to 123 full-length *NLR* genes with an entire NB-ARC and LRR domain. Similarly, Andolfo et al. employed cDNA RENseq to identify 124 and 221 full-length NB-LRR in two tomatoes [37]. Furthermore, taking advantage of the RENseq technique to enrich pattern recognition receptors (PRRs) [38], NLR and other ETI components could provide a better understanding of the broad and durable resistance mechanisms in *S. pinnatisectum*.

Our preliminary investigation of the resistance mechanisms in *S. pinnatisectum* provides an opportunity to explore potential novel ETI components and the effective combination of *R* genes for late-blight resistance breeding. Indeed, the employment of genetic engineering into late-blight resistance breeding programs should speed up the race against *P. infestans*. However, rational deployment of effective *R* genes and gene pyramiding can extend the durability of such resistance and offers potato growers an environmentally friendly alternative for late-blight management.

## 4. Materials and Methods 

### 4.1. Plant and Pathogen Materials

The diploid potato species, *S. pinnatisectum* (Accession PI275233) and *S. cardiophyllum* (Accession PI186548), which are resistant or susceptible to *P. infestans*, respectively, were used in this study. Both species were propagated in vitro in sterile jars containing Murashige and Skoog medium supplemented with sucrose and grown for about 6 weeks after transplanting into nutrient soil at 20–25 °C under a 14 h:10 h day/night photoperiod.

All *P. infestans* isolates were previously described [5,25] and were routinely maintained on RSA (rye sucrose agar) medium. The numerous sporangia collected from 7- to 10-day cultures were chilled with cold sterilized water at 4 °C for about 1 h to release zoospores. The zoospores were filtered through one layer of Miracloth and the concentration was adjusted to 1 × 10^5^ zoospores/mL. A highly aggressive isolate of *P. infestans* Pi21336, which overcomes the *R1*–*R11* genes of potato, was used for inoculation of *S. pinnatisectum* and *S. cardiophyllum* in the histological and transcriptomic analyses. Additionally, 12 other isolates with complex virulence spectra (Table 2) were also used for detecting the genetic resistance of *S. pinnatisectum*. The pathogenicity of the 12 *P. infestans* isolates on *S. pinnatisectum* and *S. cardiophyllum* was evaluated 3–4 days post-inoculation (dpi). The inoculation site for the 12 isolates on each leaflet was randomized. Three leaflets of *S. pinnatisectum* and 12 leaflets of *S. cardiophyllum* were used for each repetition and the experiment was repeated twice.

### 4.2. Lesion Measurement and Biomass Analysis

Pictures of diseased leaves were taken at 4 dpi, and the relative lesion sizes were measured with ImageJ software. For the qPCR analyses, samples (20 μL) were prepared by mixing 1 μL (5 ng) of gDNA and 19 μL of 2 × SYBR Green mix (Bimake, Houston, TX, USA) with the appropriate primers (Table S3) added to a final concentration of 5 μM each and water. PCRs were performed in triplicate for each biological sample, using the default relative quantification program of CFX connect Real-Time PCR System (Bio-Rad, Hercules, CA, USA). Ct values were determined with the included Bio-Rad software. The detection of real-time PCR products, calculations, and statistical analysis were performed as previously described [39].

### 4.3. Microscopic Characterization of S. pinnatisectum Infected by P. infestans

Detached-leaf inoculations were conducted as previously described [25] with some modifications. Leaflets were detached from robust growing plants in the greenhouse, placed on water-saturated filter paper in a tray, and inoculated on the abaxial side of both *S. pinnatisectum* and *S. cardiophyllum* with 10-μL droplets containing approximately 10^3^ zoospores. The inoculated leaves were kept in moisture trays sealed with plastic wrap at 18 °C in the dark for the first 12 h, and then the trays were incubated at 18 °C under a photoperiod of 16 h light and 8 h darkness. For microscopic observations, samples were obtained at 3, 6, 9, 12, and 72 hpi. Samples were stained with trypan blue staining following the protocol described by Wang et al. [40]. The experiment was repeated at least three times.

### 4.4. In planta Transient Expression of Known Avr Genes of P. infestans

To detect the *Rpi* genes known for *Solanum* spp. in *S. pinnatisectum* and *S. cardiophyllum*, 10 notable RxLR effector genes of *P. infestans*, Avr1, Avr2, Avr3aKI, Avr3b, Avr4, Avrsmira1, Avrsmira2, Avrblb1, Avrblb2, and Avrvnt1 were cloned into pDonr201, and subcloned into pmAEV using LR clonase (Invitrogen, USA) and then transformed into *Agrobacterium tumefaciens* GV3101 by heat shock treatment. The coding sequences of two resistance genes, *R3a* and *Rpi-vnt1*, were directly synthesized (Genscript Inc.,Nanjing, China) and inserted into a pmAEV vector.

Agroinfiltration was performed as previously described [25]. Briefly, *A. tumefaciens* strains were grown to an optical density at 600 nm of 0.2, and leaf panels of 6- to 8-week-old potato plants were infiltrated with the *A. tumefaciens* suspensions. The position for infiltration was randomized. Cell death was monitored from 4 to 6 days post-infiltration. The experiment was repeated three times. Analysis of each *Avr* gene consisted of five or three infiltrated leaves of *S. pinnatisectum* and *S. cardiophyllum*, respectively.

### 4.5. RNA-seq and Profiling of the Expression of NLR Genes

RNA was obtained by grinding tissue in liquid nitrogen, followed by extraction using the RNeasy Plant Mini Kit from Qiagen. The quality and integrity of purified RNA were verified with the Qubit^®^ RNA Assay Kit in a Qubit^®^ 2.0 Flurometer (Thermo Fisher Scientific, Waltham, MA, USA) and the RNA Nano 6000 Assay Kit of the Agilent Bioanalyzer 2100 system (Agilent Technologies, Santa Clara, CA, USA), respectively. A total of 1.5 µg of RNA per sample were used for the generation of sequencing libraries using the NEBNext^®^ Ultra™ RNA Library Prep Kit for Illumina^®^ (NEB, Ipswich, MA, USA) according to the manufacturer’s recommendations. The PCR products were purified (AMPure XP system) and library quality was assessed on the Agilent Bioanalyzer 2100 system. The clustering of the index-coded samples was conducted on a cBot Cluster Generation System using the TruSeq PE Cluster Kit v3-cBot-HS (Illumina) according to the manufacturer’s instructions. Finally, the library preparations were sequenced on an Illumina Hiseq platform and paired-end reads were generated. RNA sequencing data was deposited in the NCBI Sequence Read Archive (BioProject: PRJNA616420).

Reads passing the quality filter were aligned with the *Solanum tuberosum* reference genome using hisat2 2.1.0 [41] with default parameters. The number of mapped reads of each gene were counted by using featureCounts [42]. The TPM values (transcripts per million) of the genes in each library were calculated with in-house Perl script. To identify the *NLR* genes in potato, total potato proteins [43] were scanned for the NBS domain and LRR clans with hmmscan in the HMMER 3.1b2 package [44], with trusted cutoff parameters enabled (i.e., --cut_tc). Sequences containing both the NBS domain and LRR repeat were regarded as NLR candidates. The RNA-seq data were used to profile the expression of these *NLR* genes.

## Figures and Tables

**Table 1:** Comparison of infection process of *Phytophthora infestans* on excised leaves between *Solanum pinnatisectum* and *S. cardiophyllum* at different times post-inoculation.

Time/hpi*	*Solanum* spp.	Cysts (C)			Hyphae Infected (HI)		
		GC/C	A/C	HI/C	HE/HI	HS/HI	HP/HI
3	S. c	159/177	159/177	33/177	33/33	0/33	0/33
	S. p	77/94	61/94	5/94	5/5	0/5	0/5
6	S. c	162/162	155/162	134/162	109/134	25/134	0/134
	S. p	147/148	138/148	113/148	112/113	1/113	0/113
9	S. c	272/272	272/272	272/272	162/272	110/272	0/272
	S. p	119/119	119/119	116/119	109/116	7/116	0/116
12	S. c	145/145	145/145	145/145	25/145	84/145	36/145
	S. p	84/84	84/84	83/84	77/83	5/83	1/83

Hpi refers to hours post-inoculation; GC abbreviate for short germinated cysts; A is the abbreviation for appressoria formed by cysts observed; HE is short for hyphae infected in epidermis; HS is short for hyphae infected in spongy tissue; HP is short for hyphae infected in palisade tissue.

**Table 2:** *Solanum pinnatisectum* and *Solanum cardiophyllum* show distinct patterns of the phenotype challenged by different isolates of *Phytophthora infestans* with a different ability to infect the differential set of potato carrying *R1* to *R11*.

Isolate	Avirulence on R1-R11 Differential Host	Virulence Phenotype	
		*S. pinnatisectum*	*S. cardiophyllum*
1	1	+	+++
2	2, 5, 9	+	+++
3	1, 2, 4, 5, 7, 9, 10, 11	+/-	++
4	5	+/-	+++
5	1, 2, 5, 6, 7, 9, 10, 11	+/-	+
6	1, 2, 5, 6, 7, 8, 9, 10, 11	+	+++
7	7	-	-
8	1, 5, 8, 9, 10, 11	+++	+++
9	2, 5, 8, 9	++	+++
10	9	+/-	++
11	1, 7, 9, 11	-	++
12	0	+/-	++

“-” refers to HR or no lesion; “+” refers to sporadic lesion; “++”refers lesion without sporulating; “+++” sporulating lesion; “+/-” refers to sporadic lesion was visible at least once in three biological repeats, otherwise no lesion was observed.

## Supplementary Materials

Supplementary Material can be found at https://www.mdpi.com/1422-0067/21/9/3211/s1. File S1: the CPM values for mapped genes in potato genome; Table S1: Agroinfiltration of 10 *Avr* genes in *S. pinnatisectum* and *S. cardiophyllum*; Table S2: Summary of high-quality reads and matching to *Solanum tuberosum* genome; Table S3: Primers used in this study.

## Abbreviations

*Avr* gene	Avirulence gene
EBN	Endosperm Balance Number
ETI	Effector Triggered Immunity
hpi	hours post inoculation
HR	Hypersensitive Response
NLR	NBS-LRR (nucleotide-binding site and leucine-rich repeat)
PRR	Pattern Recognition Receptor
PVX	Potato Virus X
QTL	Quantitative Trait Loci
RENseq	Resistance Gene Enrichment and Sequencing Method
*R* gene	Resistance gene
Rpi	Resistance (*R*) genes to *P. infestans*
RSA	Rye Sucrose Agar
